# Pediatric thalamic incidentalomas: a retrospective analysis of their characteristics, evolution, management, and prognostic factors for progression

**DOI:** 10.1007/s00701-025-06632-2

**Published:** 2025-08-08

**Authors:** Jana Táborská, Katarína Horčičáková, Adéla Bubeníková, Jakub Táborský, Martin Kynčl, Miroslav Koblížek, David Sumerauer, Michal Zápotocký, Zdeněk Pavelka, Ondřej Bradáč, Vladimír Beneš

**Affiliations:** 1 Department of Neurosurgery, Second Faculty of Medicine, Charles University and Motol University Hospital, Prague, Czech Republic; 2 Center for Pediatric Neuro-Oncology, University Hospital Motol, V Úvalu 84, Prague, 150 00 Czech Republic; 3 Department of Radiology, Second Faculty of Medicine, Charles University and University Hospital Motol, Prague, Czech Republic; 4 Department of Pathology and Molecular Medicine, Second Faculty of Medicine, Charles University and University Hospital Motol, Prague, Czech Republic; 5 Department of Pediatric Haematology and Oncology, Second Faculty of Medicine, Charles University and University Hospital Motol, Prague, Czech Republic; 6 Department of Pediatric Oncology, University Hospital Brno and Faculty of Medicine, Masaryk University, Brno, Czech Republic

**Keywords:** Incidentaloma, Thalamus, Pediatric tumor, Follow-up, Prognostic factors

## Abstract

**Purpose:**

The increasing availability of advanced neuroimaging has led to a rise in incidental findings among pediatric patients. Management strategies include immediate surgical intervention, observation or surgery upon progression. These are influenced by imaging characteristics, lesion behavior over time, patient/family preferences, and the lesion’s surgical risks. The thalamus’s eloquent location often warrants a more conservative approach. Identifying features predictive of growth could help inform clinical decisions regarding surveillance and potential intervention.

**Methods:**

We retrospectively analyzed 44 pediatric patients with 46 thalamic incidentalomas, assessing radiological characteristics, temporal changes, and factors predictive of progression. Progression was defined as a change in size and/or new/increased contrast enhancement. Prognostic factors for progression (demographics, initial tumor volume, extension beyond thalamus, changes in enhancement, margin characteristics) were assessed for significance.

**Results:**

Of 46 incidentalomas, 40 were followed longitudinally. Nine incidentalomas (22.5%) showed regression, while nine (22.5%) progressed. The average time to regression was 947 days, and to progression, 516 days. Three patients underwent biopsy due to progressive changes; each was diagnosed with low-grade glioma. Statistical analysis revealed that initial incidentaloma volume, extension beyond the thalamus, and contrast enhancement were significantly associated with progression (*p* = 0.025, *p* < 0.001, and *p* = 0.001, respectively).

**Conclusions:**

Most pediatric thalamic incidentalomas are small, stable, and likely low-grade. However, approximately one-fifth exhibit progressive features, warranting prolonged surveillance. Larger initial volume, extra-thalamic extension, and contrast enhancement are significant predictors of progression and may justify earlier intervention. Individualized management remains essential, balancing natural history with surgical risk.

## Introduction

The growing availability and widespread utilization of advanced neuroimaging techniques in recent years have led to a higher detection rate of incidental findings in pediatric patients undergoing imaging for unrelated indications. Reported frequencies of such incidental findings range from 10 to 25%, with neoplastic lesions comprising approximately 0.2% of all cases [14, 19, 25]. The term “incidentaloma” refers to a space-occupying lesion with radiological characteristics suggestive of a tumor, discovered in the absence of clinical symptoms attributable to its anatomical location [29, 33]. The diagnosis of an incidentaloma presents a clinical management dilemma. Although the natural history of these lesions is generally considered to be benign and most remain clinically insignificant, malignant transformation has been documented in the literature [42]. Notably, pathological diagnoses of medulloblastoma and atypical teratoid/rhabdoid tumor have been reported following surgical resection of lesions initially identified as incidentalomas [22].

Treatment options thus vary from immediate surgical treatment (biopsy or resection), watchful waiting followed with surgery upon progression, or extended observation [20, 22, 23, 42, 49, 50]. The decision to pursue active treatment takes into account several important factors, including the radiological appearance, changes over time, patient and parental anxiety and preferences, and, perhaps most importantly, the perceived surgical challenges based on the incidentaloma’s location. Comparing the anticipated surgical risks to those associated with conservative management is a highly individualized process. However, the eloquent location of the thalamus and its surrounding structures generally favors a more cautious, less aggressive approach. Identifying thalamic incidentalomas with growth potential can aid clinical decision-making regarding the frequency of follow-up imaging and the potential need for invasive intervention.

We present a series of 44 patients with 46 thalamic incidentalomas, aiming to further characterize their radiological features, changes in appearance over time, and to identify prognostic factors for tumor progression.

## Patients and methods

### Patient selection

Patients were identified from the following sources: 1) radiology reports of pediatric patients examined between 2005–2024 were searched for the keywords “thalamus” or “thalamic”; 2) institutional neurooncological database was searched for thalamic tumor location; 3) notes from weekly neurooncological tumor board meetings were reviewed for keyword “thalamus” or “thalamic”. Inclusion criteria were: 1) thalamic location of the lesion and radiological appearance suggestive of a tumor; 2) age less than 19 years at the time of initial magnetic resonance imaging (MRI); 3) asymptomatic lesion; 4) no known cancer-predisposing syndrome (e.g. neurofibromatosis type I/II, tuberous sclerosis, Li-Fraumeni syndrome). Clinical notes were used to determine symptomatology leading to the MRI and to ascertain other inclusion criteria. A lesion was considered asymptomatic if its anatomical location or mass effect could not explain the presenting symptoms. Headaches were considered unrelated if the incidentaloma was small, parenchymal, without significant mass effect, not causing hydrocephalus, and without dural attachment [22, 23]. Patients presenting with epilepsy were included if no hydrocephalus was present and the incidentaloma was confined to the thalamus [22, 23]. Patients with hydrocephalus were included if no aqueductal stenosis was present. Identified patients were screened by two independent reviewers (a neurosurgeon and a neuroradiologist, both with more than 20 years of experience) for definite inclusion; any discrepancies were resolved through consensus.

### Study variables

Following inclusion, the following data were collected: patient demographics, reason for imaging, symptomatology, radiological characteristics, management strategy, and clinical changes observed during follow-up.

Anatomical location of the incidentaloma was categorized into the following locations: anterior, medial (abutting the third ventricle), lateral (close to the internal capsule), pulvinar, thalamopeduncular, and thalamic body [23]. Eventual extension outside the thalamus was also noted. Radiological characteristics included appearance on T1-weighted imaging (T1WI), T2-weighted imaging (T2WI), Fluid-Attenuated Inversion Recovery (FLAIR), and diffusion-weighted imaging. The appearance was compared to the ipsilateral caudate nucleus and classified as hypo-, iso- or hyperintense. Contrast enhancement was also noted. Incidentaloma volume was calculated by the ABC/2 method [21] if its shape was regular and no thin-slice MRI was available. In more complex incidentalomas or where thin-slice MRI was available, volumetry was performed using 3D Slicer software [11]. The presence of edema and the character of the border were also noted. Changes during follow-up were noted and classified as regression, progression, or stability. These included changes in size, signal intensity, or contrast enhancement; progression was defined as any enlargement in size and/or new or increased contrast enhancement. Time to radiological change was recorded in days. Two independent reviewers (see above) analyzed each MRI exam and extracted data into a standardized form. An initial joint analysis of five patients was performed to ascertain reproducibility. Any discrepancies were resolved through consensus.

In patients treated surgically, the indication for surgery, time from initial diagnosis, extent of resection, histology, molecular alterations, and additional therapy were noted.

### Statistical analysis

In order to summarize patient and imaging characteristics, descriptive analysis of demographic, clinical, and radiological data was performed. Chi-square test, Fisher's exact test, and two-tailed t-test were used to evaluate the significance of differences between subgroups (progressive tumors vs. regressive/stable tumors) as appropriate. The tested variables included patient gender and age, initial incidentaloma volume (in mm^3^), presence of edema, border character, and initial extension beyond the thalamus. A *p*-value < 0.05 was considered significant.

## Results

### Patient characteristics

In total, 44 patients (26 boys and 18 girls) met the inclusion criteria. Of these, 42 were identified from radiology reports and one was from the neurooncological database, and one from tumor board meeting notes. Age at diagnosis ranged from 6 days to 18.4 years, with a mean of 10.7 years. In the first decade (2004–2014) of the study, 15 patients were diagnosed; in the second decade (2015–2024), 29 patients were diagnosed. The main reason for MRI indication was non-specific headache (*n* = 17), diagnostic work-up for other conditions such as congenital abnormalities (*n* = 7) or epilepsy (*n* = 6), follow-up for implanted shunt unrelated to the thalamic lesion (*n* = 4), eye symptomatology such as decreased visual acuity (*n* = 4) or other reasons (e.g., collapse, trauma, failure to meet developmental milestones) (Table 1).

**Table 1:** Main characteristics of patients included

Patients	44
Male: Female	26: 18
Age, mean (range)	10.7 years (6 days—18.4 years)
Reason for MRI (%)	
*Headache*	17 (38.6)
*Diagnostic work-up for unrelated condition (congenital abnormalities, suspected neurofibromatosis)*	7 (15.9)
*Epilepsy*	6 (13.6)
*Shunt follow-up*	4 (9)
*Ocular symptomatology (decreased visual acuity, strabismus)*	4 (9)
*Other (collapse, trauma)*	6 (13.6)
Follow-up mean (range); months	51 (5.5—182)
Active approach (%)	3 (6.8)

### Incidentaloma characteristics

**Fig. 1:**
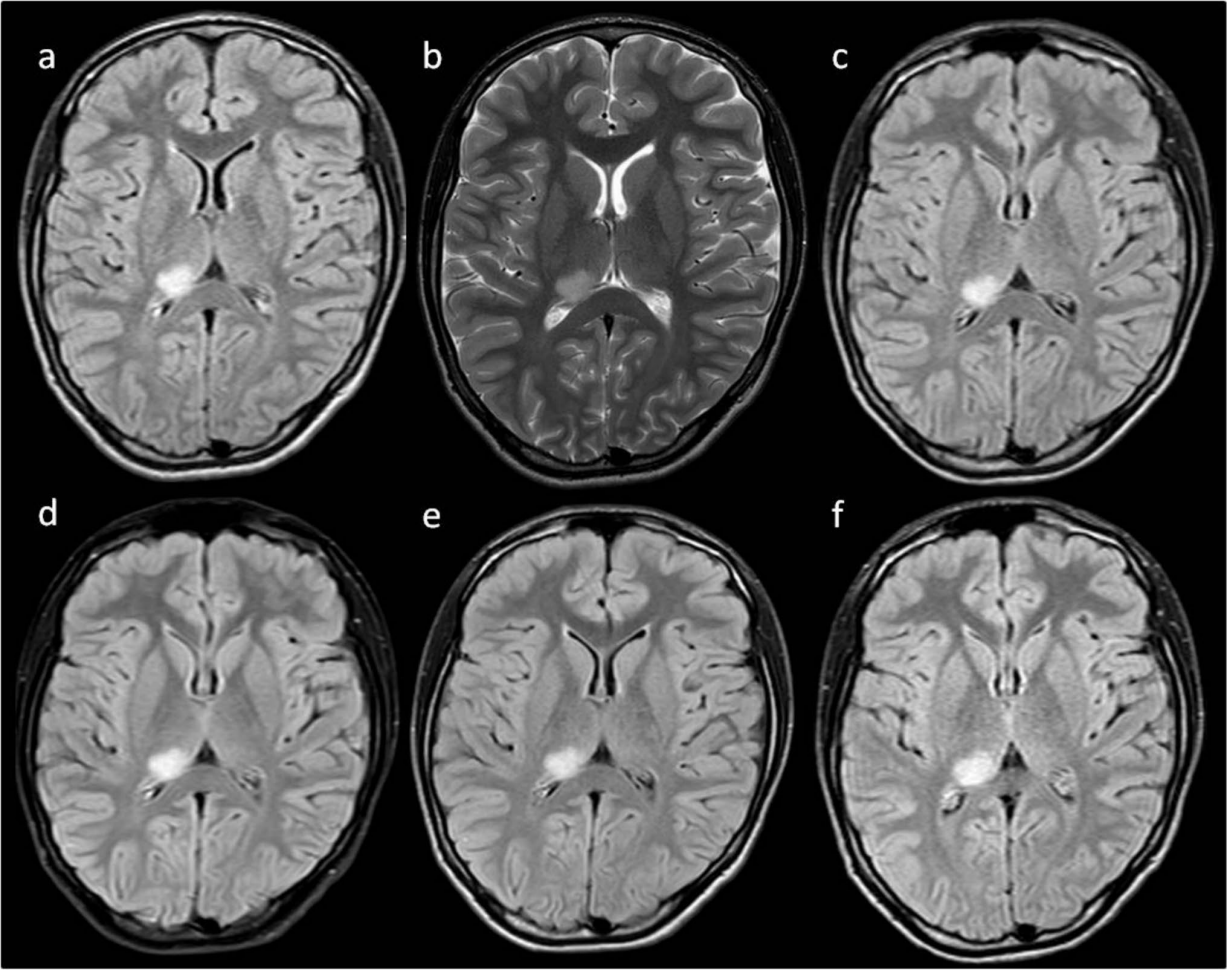
Example of a stable incidentaloma. A 15-year-old boy was examined for headaches. A hypersignal lesion on FLAIR (**a**, **c**, **d**, **e**, **f**) and T2 (**b**) images in the right posterior thalamus was diagnosed and remained stable for the duration of a follow-up of 29 months (**c**-**f**)

**Fig. 2:**
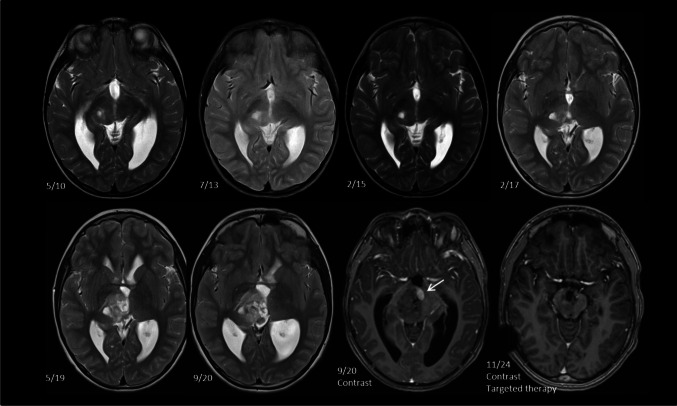
Example of a growing incidentaloma. A boy followed for post-infectious hydrocephalus was diagnosed with a thalamic incidentaloma at the age of 2 years 7 months in 5/2010. The incidentaloma showed a slight increase in size in the next 7 years. However, it dramatically increased in size in 5/2019 and 9/2020, showing areas of contrast enhancement (arrow). Following biopsy and pathological diagnosis of low-grade glioma, targeted therapy was initiated

Overall, 46 incidentalomas were diagnosed. Of these, 40 had at least two MRIs performed and were included in the longitudinal analysis. Two patients had two clearly distinct bilateral thalamic tumors, and one patient presented with a diffuse tumor involving both thalami. In total, 23 were located on the left side and 18 on the right side. The most common incidentaloma location was the pulvinar (*n* = 24), followed by medial location (*n* = 10). Initially, 7 incidentalomas extended beyond the thalamus into the third ventricle (*n* = 4), lateral ventricle, optic tract, and dorsal mesencephalon (*n* = 1 for each). Typical examples are provided in Figs. 1 and 2. The mean initial volume was 1602 mm^3^ (range 4 mm^3^—29,400 mm^3^). Initial signal characteristics are provided in Table 2. Incidentalomas were generally best appreciated on T2WI or FLAIR images. None exhibited edema or an area of restricted diffusion. Well-defined borders were identified in 16 (34.8%) cases, and 5 (10.8%) exhibited contrast enhancement on initial MRI.

**Table 2:** Main characteristics of included incidentalomas

Incidentaloma characteristics	n (%)
Location	
Pulvinar	24 (52.2)
Medial	10 (21.7)
Anterior	4 (8.7)
Body	4 (8.7)
Lateral	2 (4.3)
Thalamopeduncular	1 (2.2)
Diffuse	1 (2.2)
Extension	
Thalamus only	39 (84.8)
Third ventricle	4 (8.7)
Lateral ventricle	1 (2.2)
Optic tract	1 (2.2)
Dorsal mesencephalon	1 (2.2)
Initial tumor volume mean (range); mm^3^	1602 (4—29,400)
MRI signal characteristics	
T1WI hypointense	22 (50)
T2WI hyperintense	44 (95.6)
FLAIR hyperintense	42 (91.3)
Restricted diffusion	0 (0)
Edema	0 (0)
Contrast enhancement	5 (10.8)
Well-defined borders	16 (34.8)

### Follow-up and incidentaloma development

**Fig. 3:**
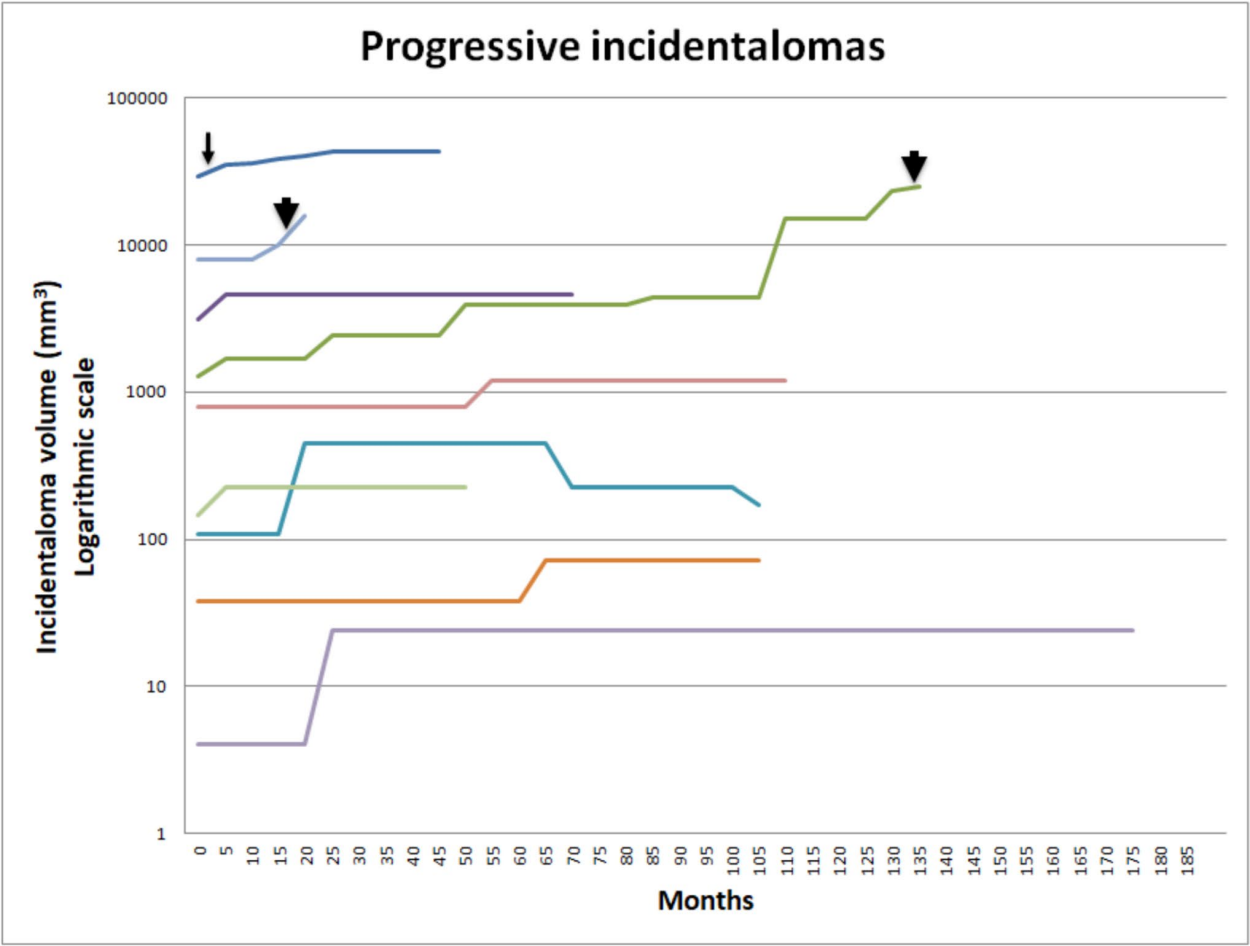
Development of incidentaloma volume that enlarged at any time during follow-up. Development of incidentaloma volume that enlarged at any time during follow-up. Logarithmic scale on the Y axis. Actively managed tumors are depicted with an arrow (biopsy only) or arrowhead (biopsy and molecular targeted therapy) at the time of intervention

During the follow-up period (mean duration: 51 months), 9 incidentalomas (22.5%) showed signs of regression: 5 demonstrated reduced signal intensity on T2WI/FLAIR images, 3 decreased in size, and 1 showed both reduced signal intensity and size. In contrast, 8 incidentalomas (20%) exhibited signs of progression: 4 increased in size, and 4 showed both an increase in size and contrast enhancement. One initially increased in size but subsequently decreased (Fig. 3). The remaining 22 incidentalomas exhibited no change during the follow-up period. The mean time to any regression was 947 days (range 79–2271 days), whereas the mean time to any progression was 516 days (range 70–1612 days). The mean volume of maximal tumor regression was 400 mm^3^ (range 9–2552 mm^3^), whereas the mean volume of maximal tumor progression was 6000 mm^3^ (range 20–23826 mm^3^). During the follow-up period, three patients underwent tumor biopsy after 134.6, 16, and 2 months of observation. The reason for the active approach was continuing size progression and increased contrast enhancement. The definitive diagnosis was low-grade glioma in all cases. One patient harboured *BRAF T599dup* mutation and two *BRAF V600E* mutation; both patients with *BRAF V600E* received targeted molecular therapy using a combination of dabrafenib plus trametinib. The remaining patients remain under observation. No clinical progression or new symptoms attributable to the thalamic incidentaloma were observed in any patient during follow-up.

### Statistical analysis

For the sake of statistical analysis, the one incidentaloma showing initial progression and then regression was analyzed in the progressive group. Of the tested variables, initial volume (*p* = 0.025), initial extension beyond the thalamus (*p* < 0.001), and change in contrast enhancement (*p* = 0.001) showed significant differences between progressive tumors and regressive/stable tumors. However, we were unable to determine the minimum lesion volume necessary for an incidentaloma to be susceptible to progression (Fig. 3). The other tested variables (patient demographics and margin character) did not reach significance. Only progressive incidentalomas were actively managed (*p* = 0.008) (Table 3).

**Table 3:** Statistical analysis comparing demographic and radiological variables of progressive (P) incidentalomas to combined regressive (R) and stable (S) incidentalomas

	Progression (P)	Regression (R)	Stable (S)	R + S	p; P x R + S
n	9 (22.5%)	9 (22.5%)	22 (55%)	31 (77.5%)	
Male	6	6	15	21	
Female	3	3	7	10	0.951
Age (mean)	10,4	8,4	11,6	10,8	0.898
Initial volume (mean; mm^3^)	4750	541.8	865	764.2	**0.025**
Extension beyond thalamus	5	0	2	2	**0.001**
Thalamus only	4	9	20	29	
Change in enhancement	4	0	0	0	**0.001**
No change	5	9	22	31	
Well-defined margin	3	6	7	13	0.642
Unclear margin	6	3	15	18	
Active management	3	0	0	0	**0.008**

## Discussion

Reports of imaging characteristics and management of pediatric thalamic incidentalomas have been scarce in the literature [2, 23]. In this context, our study has the potential to provide additional insights into natural history, incidentaloma development, and prognostic factors associated with progression. The main findings of this study can be summarized as follows: 1) most thalamic incidentalomas are small and clinically inconsequential; 2) about one in five showed signs of progression; 3) the same proportion showed signs of regression; 4) growth was associated with larger initial volume, initial extension beyond the thalamus, and changes in contrast enhancement.

### Symptomatology

No consensual definition of an incidentaloma is agreed upon in the literature. However, symptomatology unexplained by its location is generally required. Our most common presenting symptom—headache—is rather typical of incidentaloma studies [1, 2, 22, 23, 41, 49, 50]. We carefully reviewed patients examined for headaches and included those with small parenchymal incidentalomas without perifocal edema or significant mass effect. Dural attachment was also absent in all our patients. Patients with hydrocephalus were included only if the diagnosis preceded incidentaloma detection, or if concurrent, only in the absence of aqueductal stenosis was evident or when other etiology was confirmed. Additionally, patients followed after earlier shunt implantation without incidentaloma diagnosis prior to hydrocephalus treatment were also included. In the absence of additional symptoms, these newly developed lesions cannot be considered symptomatic.

Other diagnostic work-ups led to brain MRI for exclusion of other congenital abnormalities, neurofibromatosis (excluded in all patients), or epilepsy. Due to its rich connections to the cortex and vice versa, the thalamus is often involved as a relay in epileptic circuits [3, 8]. However, no broad consensus supports the thalamus as an epileptogenic center [27]. Thus, consistent with other studies, we included these patients as well [2, 23].

### Imaging features

Similarly to others [2, 23], most of our incidentalomas were hyperintense on T2WI and FLAIR imaging, hypointense on T1WI, located in the posterior thalamic regions, and showed contrast enhancement in only a small portion of patients. No lesion with restricted diffusion was identified, in accordance with Alves´s study [2]. All these features point to low-grade histology, and this was confirmed in three cases. Obviously, no confirmation is available in the non-biopsied cases; however, a benign clinical course and regression in one-fifth of patients during the follow-up period tends to argue against malignancy. Imaging characteristics suggestive of high-grade pathology include changes in the pattern of contrast enhancement, growth, and restricted diffusion. Indeed, the former two features led us to an active approach to exclude malignancy.

Although no lesions in our cohort demonstrated restricted diffusion, the interpretation of ADC values in very small thalamic lesions remains technically challenging due to partial volume averaging, ROI placement variability, and spatial resolution limits [40]. While ADC values can support lesion characterization, they must be interpreted within the broader clinical and radiological context, including morphology, stability, and absence of mass effect or enhancement [2, 15]. Furthermore, although not included in this retrospective analysis, future studies incorporating diffusion tensor imaging (DTI) and fractional anisotropy (FA) may further refine the ability to distinguish neoplastic from non-neoplastic thalamic lesions, as previously suggested [16, 37].

In order to further distinguish between low- and high-grade pathology or malignant transformation, other imaging modalities have been applied, including positron emission tomography (PET), perfusion imaging, or magnetic resonance spectroscopy (MRS) with varying degrees of success. García-Gómez et al. showed that MRS can differentiate glioblastoma, meningioma, metastasis, and low-grade glial tumors with about 90% accuracy in a multicenter study [13]. Vincente et al. confirmed the technique's high diagnostic accuracy in pediatric brain tumors across 10 centers, especially when using two echo times. Automated processing and pattern recognition make MRS a reliable, noninvasive diagnostic tool [46]. Perfusion imaging with MR or computer tomography further enhances tumor characterization by assessing vascularity, aiding glioma grading, treatment planning, and monitoring. This in vivo approach reveals tumor physiology and hemodynamics, complementing conventional imaging and histology [18]. Pirotte et al. found that PET imaging surpasses MR in sensitivity and specificity for detecting tumor tissue and malignancy in children with incidental brain lesions. As a complementary functional tool, PET can improve surgical planning, building on adult brain tumor experience and current guidelines [34]. Preliminary pediatric applications of PET demonstrate its potential to guide surgical decision-making through metabolic data, establishing functional neuroimaging as a valuable tool in pediatric neuro-oncology [34].

### Malignant transformation

Although very rare in the pediatric population, cases of malignant transformation have been reported in recent literature [7, 17, 47, 50], including in incidentalomas [43]. Due to the rarity of this phenomenon, no clear imaging characteristics associated with malignant transformation have yet been identified. Predisposing factors such as previous photon radiation [32] or cancer predisposition syndrome (neurofibromatosis) have shown an association. Pediatric patients harboring molecular aberrations, such as alterations in *CDKN2A,* or *TP53*, have an increased risk of malignant transformation [12, 28]. No cases of malignant transformation were identified in our cohort, although the previously stated caveats regarding unverified histology and relatively short follow-up need to be kept in mind. Similarly, patients with cancer predisposing syndromes were excluded, introducing understandable selection bias.

### Proportion of change

The reported proportion of progressive radiological change in incidentaloma (22.5%) aligns with other reports of pediatric brain incidentalomas [22, 23, 49, 50], a ratio further supported by a large literature review [20]. By contrast, in a large study of 171 pediatric thalamic incidentalomas, Alves et al. found only 6% of enlarging lesions [2]. This study included all non-specific localized thalamic signal abnormalities, excluded patients with mass effect and/or contrast enhancement, and no patient underwent pathological verification. A wide range of diagnoses, including hamartomas, dysplastic changes, inflammation, or epilepsy-associated signal changes, could thus have been included. Bias introduced in this way would dilute the actual growth rate [2]. The lack of progression in the majority of our cases does not exclude tumor etiology; in a large study by Wissoff et al. residual low-grade gliomas smaller than 1.5 cm3 remained progression-free during 8 years in 56% of patients [48].

The mean time to progression observed in our study was 516 days (17.2 months), reflecting a comparatively prolonged progression period for incidentalomas relative to previously published findings. Kozyrev found marked radiological changes after a mean time of 9.7 months [23], Alves after a median of 430 days (14.3 months) [2], and Zazoue after a median of 25 months [50]. Unfortunately, patient selection and methodology for all these studies is different, direct comparison is therefore not possible. However, looking at our progressive tumors (Fig. 3), only 3 showed repeated progression, whereas the others exhibited only one period of growth followed by a period of stability. The continuously growing tumors eventually underwent biopsy/targeted therapy in comparison to the progressive/stable incidentalomas, where the strategy of watchful waiting is continued. The obvious implication concerns the interval of surveillance MRI. Based on our data and in accordance with others [20, 50] we suggest a 3, 6 months, 1, 2, 3, and 5 years surveillance protocol with contrast application after initial detection of thalamic incidentaloma, barring progression or symptoms development.

### Factors predicting progression

Studies in adults have shown that incidental low-grade gliomas are typically progressive tumors, characterized by constant imaging growth with a velocity of diametric expansion of approximately 3.5 mm per year—similar to the growth rate observed in symptomatic low-grade gliomas [26, 31, 35]. In contrast, studies examining pediatric patients suggest a more favorable natural history for suspected benign incidentalomas, with rare instances of progression or malignant transformation. Some reports even describe the spontaneous regression of these lesions. This starkly contrasts with adult data, where progression of low-grade lesions is common and malignant transformation occurs in up to 50% of cases [41].

In our study, we identified greater initial tumor volume, contrast enhancement at any time point, and initial extension beyond the thalamus as factors associated with progression. To our knowledge, these findings are novel within the incidentaloma literature. Previous authors have either not reported prognostic factors for tumor progression [2, 22, 23, 49] or found no significant association between progression and imaging features such as size, contrast enhancement, or edema [50]. Notably, Zaazoue et al. reported that lesion size, location, multiplicity, new-onset symptoms, contrast enhancement, and edema were not predictive of radiologic progression [50].

It remains to be seen how these findings will influence future management strategies for pediatric incidentalomas.

### Management strategy

Contrary to other brain locations, the thalamus represents a surgically more challenging area, where the risk of permanent and devastating neurological deficit has tended to discourage surgeons from pursuing radical surgeries in the not-so-distant past. However, advances in imaging protocols, intraoperative monitoring, microsurgical techniques, and image guidance have substantially contributed to acceptable morbidity rates in symptomatic patients in recent series [4, 6, 9, 24, 39, 44, 45, 51]. In these patients, frequently presenting with hydrocephalus or focal neurological deficit, the natural course of the disease outweighs the risks of surgery. The greater size of symptomatic tumor also allows the surgeon to access the lesion relatively safely through well-defined anatomical corridors and approaches [4, 5, 10, 30, 36, 38, 39]. The goal is to minimize transgression of the neural tissue, or to approach the tumor where it extends beyond the thalamus [4]. Obviously, these surgical “advantages” are not applicable in incidentalomas. The typical incidentaloma is small, located deep in the thalamus, without reaching its surface, and surgical risks are therefore deemed unacceptable in an asymptomatic patient without worrying MRI characteristics. Contrary to adult patients, where resection of low-grade gliomas has been proven to prolong survival and reduce rates of malignant transformation, no clear evidence supporting this approach in children is available. Thus, due to anticipated surgical risks associated with eloquent thalamic location, observation in an asymptomatic patient is a very reasonable management strategy. Alternatively, in tumors presenting with possible malignant attributes (restricted diffusion, growth, changes in enhancement), observation should be close and frequent, and the threshold for active management very low. Similarly to our series, these features have been associated with surgery during follow-up in several studies [22, 23, 50], to exclude malignancy or to provide actionable molecular targets (Fig. 2). Furthermore, a literature review found a 15% rate of high-grade histology among incidentalomas [20]. The decision for surgical treatment is therefore highly individualized and takes into account the mentioned MRI characteristics, anticipated surgical risks, and patients'/parents'preferences.

### Limitations

Interpreting our results, the usual limitations of retrospective studies need to be acknowledged. Although we tried thoroughly to include all possible patients, some may have inadvertently escaped our attention, and bias cannot be completely excluded. Also, the number of patients is relatively modest, precluding more robust statistical analysis. The study period encompasses 20 years, during which time MRI protocols have evolved, and patients were not examined on the same scanners. Ideal reproducibility of the MRI exams cannot be ascertained this way. We tried to mitigate this limitation by having two independent reviewers with extensive experience assess the images. Additionally, small incidentalomas could not be accurately measured volumetrically on thicker-slice MRI scans, so the ABC/2 method was used to calculate tumor volume. We believe that applying this method consistently within each patient minimizes potential bias. Another limitation is the relatively short follow-up, which needs to be acknowledged. Prolonged observation would certainly reveal other longitudinal changes in size or image characteristics; these would lead to a more active approach in selected progressive patients or to surveillance discontinuation in regressive tumors.

## Conclusion

The majority of pediatric thalamic incidentalomas are small and clinically inconsequential and can safely be presumed to be low-grade histology. However, about a fifth will eventually show progression in size or contrast enhancement, thus extended surveillance is mandatory. Greater initial incidentaloma volume, initial extension beyond the thalamus and contrast enhancement were identified as predictive factors for progression and should be taken into account when considering an active approach. However, management decisions should be made on an individualized basis and must carefully compare favorable natural history to anticipated surgical risks.

## Data Availability

No datasets were generated or analysed during the current study.

## Abbreviations

FLAIR: Fluid-Attenuated Inversion Recovery
MRI: Magnetic resonance imaging
MRS: Magnetic resonance spectroscopy
PET: Positron emission tomography
T1WI: T1-weighted imaging
T2WI: T2-weighted imaging
